# Teleworking: does it make workers healthier and productive? A cross-sectional study on a Southern European population

**DOI:** 10.1186/s12889-024-19481-y

**Published:** 2024-07-20

**Authors:** Julian Perelman, Florentino Serranheira, Twork4Health Group, Filipa Castanheira, João Filipe Raposo, Pedro Aguiar, Pedro Neves, Sara Ramos, Sónia Dias, Teresa Maia, Pedro Laires

**Affiliations:** NOVA National School of Public Health, Public Health Research Centre, Comprehensive Health Research Center, NOVA University of Lisbon, Lisbon, Portugal

**Keywords:** Telework, Health, Productivity, Well-being, Portugal

## Abstract

**Background:**

Teleworking (TW) has recently shifted from a marginal into a common practice. Yet, concerns have been raised regarding potential work-health negative effects, related to the reduced socialization, and extended working hours with computers at home, possibly offset by reduced commuting time or better individual work-life balance. This paper aims at describing the influence of TW on health, well-being, and productivity perceptions, and how this is shaped by TW conditions.

**Methods:**

We collected data from workers of 25 companies that exert their activity in Portugal. Data were completed with a representative sample of workers who regularly participate in surveys (total *N* = 1,069). We applied an on-line questionnaire from September the 1st 2022 to December the 1st 2022. We performed a simple descriptive analysis of each variable. Then, we analyzed the relationship between TW conditions and self-reported health, and between TW conditions at home and productivity, using logistic regression models.

**Results:**

We observed a high prevalence of self-perceived health worsening (15.9%), mostly among those with poor TW conditions. Most teleworkers enjoyed favorable TW conditions, despite limited company support. Relevant changes were observed in lifestyle factors, towards more smoking (5.5%), alcohol drinking (4.5%), and worse diet (10.1%). Two thirds reported enhanced productivity. A statistically significant relationship was observed between inadequate TW conditions, health deterioration, and lower productivity. A 6.0% point (pp) increased risk of productivity worsening was observed when employees faced at least one inadequate condition at home (no private working place at home, inadequate heating, artificial light, or absence of well-being at home). The risk of health deterioration increased by 12.9 pp when facing at least one of these inadequate conditions, and by 6.3 under hybrid TW, compared to one or two days of TW.

**Conclusions:**

Most teleworkers highlighted a positive perspective about teleworking. Yet, TW conditions are not favorable for all workers, with consequences on health, well-being, and productivity, suggesting that further support is needed for teleworkers to protect their health at home, and reach its maximum benefit.

**Supplementary Information:**

The online version contains supplementary material available at 10.1186/s12889-024-19481-y.

## Introduction

The recent pandemic of SARS-COV-2 (COVID-19) has led authorities of all European Countries to impose severe social distancing measures, transforming teleworking (TW, “working from a variety of alternative locations outside of the central office” [1] using technology to interact with others) from a marginal practice into a common practice [2, 3]. In 2008, less than 8% of employees were teleworking ‘sometimes’ or ‘usually’ in the EU Member States [4]. This share gradually increased over the years, reaching 11% in 2019, just before the pandemic, which led to a sharp increase up to 19% in 2020 and to 22% in 2021 [4]. This growth was supported by digitalization, which was considered by the European Commission (EC) as a major driver of economic recovery [4].

Authors have long identified, from theoretical and empirical viewpoints, that the well-being consequences of TW are ambiguous, a conclusion referred as the “telecommuting paradox” [5]. Gajendran and Harrison highlighted the increased and beneficial autonomy under TW in terms of work location, scheduling, and means of work; they also find a lower work-family conflict related to the better synchronization between the work and family domains, but also the impoverishment of work relations, leading to worse communication, more isolation, and reduced career opportunities. Other authors argued that the work-life balance might be deteriorated by blurred work-life boundaries and increasing working hours [1]. The combination of these three psychosocial factors (flexibility, work-life imbalance, personal interactions) leads to unclear consequences in terms of job satisfaction, stress, performance, or turnover [1].

Meanwhile, workers and employers have more recently clarified some of these advantages of TW, paving the way for its expansion beyond the pandemic. For example, some of the perceived benefits included better concentration at work and reduced commuting time [6]. On the negative side, the detrimental consequences were newly highlighted of relational impoverishment, extended working hours, or blurred work-life boundaries [7].

Meanwhile, the balance between positive and negative factors are not uniform, heavily depending on TW conditions. First, the TW physical conditions are relevant, such as the possibility to benefit from a private workspace and adequate equipment and, more generally, from an ergonomic workplace (as an ergonomic office equipment, adequate light and heating, noiseless environment, etc.) [8–10]. Based on these arguments, the EC notes that TW benefits may be unequally distributed. Indeed, workers may have, or not, a home office and access to ergonomic equipment, neither to the best information and communications technology (ICT) at home. Adverse conditions at home may contribute to the relationship between TW and a poor physical health status. Second, the advantages of TW may be hindered by pressing job demands, e.g., long working hours and low autonomy, especially if worsened by higher isolation, decreased inter-personal contacts, or caring demands at home [10, 11]. These aspects may be also aggravated in case of poorly supportive leadership, low caring culture, and insufficient technology support [12]. Hence, these psychosocial factors related to TW conditions may contribute to the adverse effect of TW on mental health (stress, anxiety, depression). By contrast, the adoption of job crafting strategies was viewed as a major tool to mitigate these disadvantages, such as learning new ICT skills, online socialization with colleagues, or reducing or optimizing work demands through well-defined day structures and reduced work availability [12].

From a fully empirical perspective, many studies have long demonstrated the relationship between working conditions, living arrangements, health, and well-being [13–15]. Teleworking, however, is a new reality that mixes these aspects, whose link to health and safety at work is, as expected, based on few and contradictory evidence [16]. A rapid literature review from 2020 identified 23 studies, most of them on mental-health related impacts [17]; the focus on mental health is no surprise, since the hypotheses about TW effects relate to flexibility, work-family conflict, and isolation [5]. Studies evaluated effects on stress, well-being, quality of life and depression, finding generally mixed results, mirroring the ambiguity of theoretical expectations. Differences in organizational responses and support were identified as important contributors to either increasing or mitigating negative health outcomes. Regarding physical health, authors suggested potential effects of TW related to more sedentary behaviours and increased screen time, leading to musculoskeletal disorders; unhealthy diet or increased alcohol consumption; or greater stress associated to a higher risk of heart diseases [9, 18]. A recent systematic review of TW effects on musculoskeletal disorders found an increased risk of lower back and neck pain, associated with poor ergonomic home conditions [19]. Finally, a recent review of 19 studies found an increase in TW-related stress in most cases [20].

From the company’s perspective, understanding how TW affects workers’ health and safety is also a major issue. On the one hand, there is evidence that interventions introducing flexible work and scheduling changes are effective in improving workers’ well-being, measured in various ways [21] On the other hand, studies before and during the pandemic found favorable effects of TW on productivity [22, 23] and a linkage between flexible work arrangements and productivity [24, 25], but evidence remains scarce. Meanwhile, no study so far has identified the role of health and well-being in mediating the link between TW and productivity.

Noticeably, most EU Member States have started to regulate TW, including Portugal, driven by the emergency of a new, large, and challenging phenomenon. Yet, decisions have been made in urgency without being, in most cases, supported by sound evidence.

This paper has two main objectives, based on two key hypotheses. First, we postulate that self-perceived health and productivity are positively impacted by the frequency of TW up to a certain point, i.e., when TW intensity is too high, the negative consequences of isolation outweigh the possible benefits. Second, we postulate that self-perceived health and productivity are related to physical conditions at home, and that these conditions mitigate the effect of the TW frequency [26].

## Methods

This study is reported following the Strengthening the Reporting of Observational Studies in Epidemiology (STROBE) reporting guideline.

### Study design

This is a cross-sectional study, based on an on-line questionnaire. The survey was submitted to the Ethical Commission of the Nova Medical School, which granted authorization on the 1st of July 2022 (Final Declaration 81/2022/CECFM).

### Setting

The participants were recruited and participated in the survey from September the 1st 2022 to December the 1st 2022. Given the cross-sectional nature of the study, there was no follow-up, and the questionnaire was entirely performed on-line.

### Participants and sample size

We recruited companies that exert their activity in Portugal with at least 5% employees working remotely. To do so, we got the support of the Portuguese Social and Economic Council (CES), which groups many companies’ associations and unions and is devoted to social conciliation and to the elaboration of recommendations to policy makers. A total of 25 companies participated in the study, including large size companies with high visibility. From the participating companies, we reached an end sample size of 568 participants (by 24th of November 2022).

In addition, we used a sample of 500 workers, who regularly participate in surveys by a private multinational survey company (Growth for Knowledge, Gfk). This sample has been recruited through online platforms. This sample, although recruited through voluntary and paid participation, is quite alike the Portuguese population eligible for TW in terms of demographic characteristics.

The final sample included 1,068 participants.

### Data source

The on-line questionnaire was developed based on the literature (see below for sources related to specific questions/variables) and validated and complemented by a multi-disciplinary research group including an epidemiologist, an occupational health specialist, a health economist, an occupational psychologist, and clinicians specialized in various areas (e.g., psychiatry, cardiology, endocrinology, and rheumatology). The survey included the following dimensions: sociodemographic and self-reported health; physical TW conditions; physical health (including musculoskeletal symptoms and cardiometabolic changes); mental health; work engagement, absenteeism, and productivity. We describe here-below the variables constructed, based on the questions, used in the current study.

### Variables

#### Health and productivity outcomes

We used, for health outcomes perception and determinants, questions about the deterioration of the physical health condition or health behavior related to TW, which included four possible answers: “not affected by the problem”, “condition has improved”, “condition has not changed”, “condition has worsened”. The outcomes and its work determinants included general self-reported health, musculoskeletal symptoms, fatigue, sleep problems, stress, anxiety, depression, work engagement, work-family conflicts, and well-being. A question was addressed on productivity changes since TW initiation, with three answer possibilities, “improved”, “maintained”, or “worsened”. We grouped the “maintained” and “improved” categories to construct a binary variable, with a value 1 in case of worsening. This type of measurement, into these three possibilities, was inspired on the Survey on Health and Aging in Europe (SHARE), in its eight wave [27].

#### Teleworking conditions

Teleworking conditions at home were assessed through questions about private workspace, artificial *versus* natural light, adequate heating, and well-being in the working space [28]. For each question, whenever possible, a list of 5 answers were proposed, from “rarely” to “always” (e.g., we asked if the person felt privacy in the workspace). The only exception was for artificial versus natural light, for which possible answers were “yes” or “no”. These variables were recoded into binary variables, where the value 1 corresponded to the worst condition (no privacy, no natural light, no adequate heating, and no well-being in the working space). We finally created a binary variable, with a value 1 for those facing at least one inadequate working-at-home condition, and zero otherwise.

Additionally, a categorical variable was created for the TW frequency, with the categories “one or two days per week in TW”, “three or four days per week in TW”, hybrid model (undefined flexible number of TW days), and “full time TW”.

#### Covariates

The socio-demographic questions included age and sex, and highest education level (from completed 4th degree, primary school, secondary education, tertiary education.

### Statistical methods

We performed a descriptive analysis of each variable. Then, we analyzed the relationship between TW conditions, including TW schedules, and self-reported health, and between TW conditions at home, self-reported health, and productivity. This last analysis was performed using logistic regressions, which modeled self-reported health (binary outcome, i.e., “poor” or “very poor health” versus “fair”, “good” and “very good” health) and productivity (binary outcomes, i.e., “deterioration” versus “same or improvement”) as function of working conditions. These analyses were adjusted for age, sex, and education level, while all analyses included random company effects, to account for possible clustering. The results from logistic regressions were presented as adjusted risk differences, expressed in percentage points (or marginal effects, using an alternative term).

Finally, a model was tested with interactions between TW schedules and working conditions at home, to evaluate whether the impact of working conditions was stronger among those spending more days working at home. All analyses were performed using *Stata* statistical software (version 17.0SE, Stata Corporation, College Station, Texas, USA).

## Results

The sample included 1,069 participants. Two thirds were women, and one third were men (Table 1). The most represented age category was people aged 40–49 years old (35.7%), followed by those aged 30–39 (28.2%). The smallest category included people older than 60 (3.2%). Three quarters of our sample had tertiary education. Half of the sample worked in the distribution or retail sectors. A quarter worked in IT, consulting, or research. Other sectors represented less than 10% of the sample. A large majority of the participants worked in the private sector (90.3%) and were in a teleworking regime at the time of the survey (88.5%) (not displayed in Tables). Most teleworkers had an office at home which is not shared (78.0%), with privacy (79.9%), natural light (88.0%), and adequate heating (72.1%). A majority did not face any inadequate working condition at home, but 23.5% faced at least one inadequate working condition.

**Table 1:** Sample description

	*N*	Percentage
Sex		
Female	706	66.0
Male	363	34.0
Age groups		
< 30	163	15.5
30–39	297	28.2
40–49	377	35.7
50–59	184	17.4
> 60	34	3.2
Education		
Primary	14	1.3
Secondary	254	23.8
Tertiary	798	74.9
Activity sector		
Bank	60	6.9
Distribution	434	49.9
Consulting, IT, research	215	24.7
Education	40	4.6
Tourism	14	1.6
Industry	107	12.3
Remote work		
1–2 days	209	22.8
3–4 days	179	19.5
Undefined hybrid	337	36.7
Full time	193	21.0
Working conditions at home		
Own office	551	59.6
Privacy (fully agree/agree)	738	78.0
Natural light	814	88.0
Heating (always/very often)	666	72.2
Feeling well at home (fully agree/agree)	769	83.0
Working conditions at home		
No inadequate condition	704	76.5
At least one inadequate condition	216	23.5

**Fig. 1:**
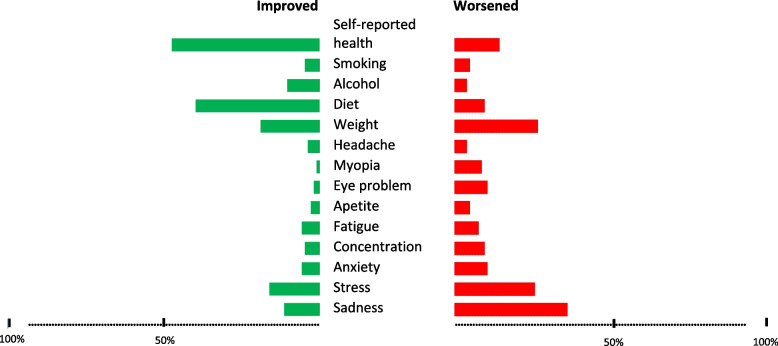
Change in health habits, physical and mental health conditions after teleworking

For the subsample of teleworkers, at the time of the survey, a total of 5.6% persons reported smoking more since working remotely (Fig. 1), more alcohol consumption (4.3%), worse diet habits (10.1%), while weight gain was the most reported lifestyle worsening (28.2%). Relevant prevalence of worsening conditions was self-reported for back pain (12.0%), neck pain (11.0%), eyes problems (11.0%), myopia (10.0%), fatigue (9.0%), appetite problems (5.4%), and headache (5.0%). More globally, 16.0% of participants reported a health deterioration.

We observed a relevant prevalence of TW-related worsening of sleep problems (11.4%), work-related stress (17.9%), anxiety (11.0%), and concentration problems (10.8%). A large proportion of people reported worsening of loneliness (39.1%) and sadness (38.4%) feelings.

**Fig. 2:**
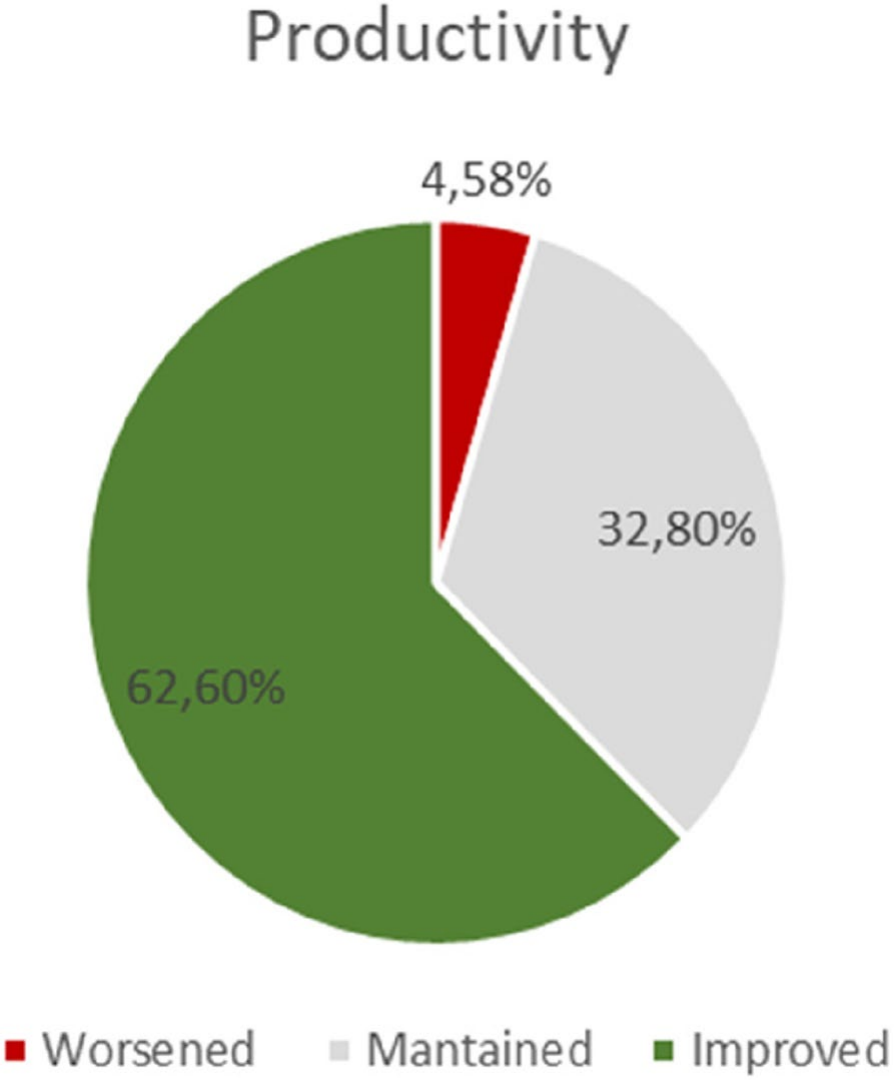
Perception on productivity after working at home

Almost two thirds of the participants reported a productivity improvement following the start of teleworking (62.6%), while only a minority reported a decline in their productivity at work (4.6%) (Fig. 2).

**Fig. 3:**
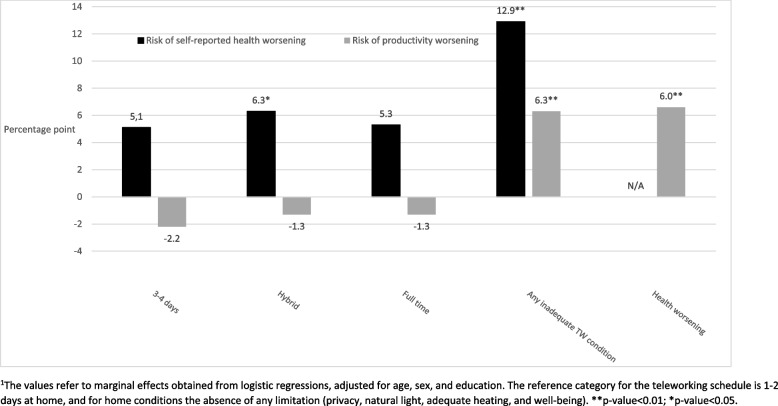
Association between working conditions and health/productivity outcomes^1^

Compared to working one or two days at home, more intense TW was associated to a higher risk of reporting poor health, and a lower risk of productivity worsening, although the latest effect was not statistically significant (Fig. 3, Supplementary Table S1). Inadequate TW conditions at home increased the risk of ill-health and productivity losses. Noticeably, people facing at least one inadequate working condition at home had a greater the risk of reporting poor health (12.9% points higher risk). This result was also observed for productivity, since having at least one inadequate working conditions was associated with a higher risk of productivity worsening (6.3% points excess risk). None of the interactions between TW schedules and working conditions at home were statistically significant (data not shown), so that working conditions at home did not modify the relationship between TW schedules and outcomes.

Finally, only 3.9% of our sample declared not willing to continue in a TW regime, for 33.5% who would like to remain in partial TW, and 62.6% in a full TW regime (not in Tables).

## Discussion

### Key findings

Most teleworkers reveal a positive perspective about this working situation, highlighted by the very high percentage of people willing to maintain this working regime. Yet, TW conditions are not favorable for all, with a substantial share of our sample reporting an inadequate TW environment. Also, around one sixth of the participants reported a health deterioration since TW, with noticeable worsening of sleep and concentration problems, anxiety, stress, loneliness, and sadness. Spending more time in TW was significantly related to a greater risk of health deterioration, but to a lower risk of productivity decline (although non-significant). A significant relationship was observed between inadequate TW conditions, health deterioration and lower productivity, suggesting that further support is needed for teleworkers to protect their health and safety at home, for TW reaching its maximum benefit. Finally, TW conditions did not mitigate the relationship between TW frequency, health, and productivity.

### Interpretation

Interestingly, the risk of health worsening increased with more time spent in TW, in comparison with one or two days. The inverse relationship has already been observed between mental and physical health and the percentage time working at home [9]. Following the framework from Gajendran and Harrison, we postulated that a small proportion of TW (one or two days) increased autonomy and flexibility, while preserving social interactions; but that this may not be the case when TW becomes the most common working practice, i.e., when isolation and poor communication become serious concerns [5]. This first hypothesis was not fully confirmed by our data. If, on the one hand, three days or more of TW was observed to be detrimental for health, on the other hand we could not compare the effect of one or two TW days with the absence of TW, since most of our sample was composed of teleworkers. The negative health consequences of more intense TW may be explained by isolation and poor communication, as described by Gajendran and Harrison [5] Other explanations may be considered, such as the observed rise, in our sample, in unhealthy behaviors, with easier access to unhealthy food or alcohol out of working premises, confirmed in other studies that also showed more sedentary behaviors [29, 30]. This aspect possibly relates to the well-known impact of context on lifestyle, which is not shaped by peer pressure (workmates) and employers’ norms when teleworking.

The beneficial effects on productivity perception have already been observed in the literature [22]. Bloom and colleagues suggest that productivity is not boosted by TW from the managers’ perspective, but well from the workers’ perspective, who produce the same work in less hours due to commute time savings [2].

To our best knowledge, the role of physical TW conditions at home has not been studied yet to explain the TW-health relationship. Studies in this field have more focused the role of living arrangements [31]. Yet, a vast literature shows that indoor environment at work affects the workers’ well-being and productivity [32]. For example, the role of some working conditions and productivity has been already established [33]; in turn, the relationship between poor health and lower productivity has also been demonstrated [34]. Our findings confirm our second hypothesis of a relationship between TW conditions and health and productivity outcomes. Based on this relationship, we may hypothesize that the inadequate working conditions at home decrease the workers’ performance through their negative effect on mental health.

Although exploratory, our descriptive analysis brings insights about mental health that we consider relevant and would deserve further specific research. Mainly, our findings confirm the worsening of mental health symptoms observed in another paper that showed increased stress [35]; however, other studies observed decreased stress [36–38], alcohol abuse and depression [39], contradicting our results. Also, other studies did not observe any significant changes of mental health symptoms [40], and a review globally confirmed mixed results [17]. Note that the results of our study are not fully comparable to earlier contributions, because our sample was mostly constituted of teleworkers, hindering a comparison to other workers, including more detailed statistical analyses. Also, our study was carried out during the post-pandemic period, which makes our findings hardly comparable to those obtained in the pre-pandemic or pandemic periods, where TW was in its first steps or justified by public health measures, respectively. By contrast, our findings are more likely to address the effective influence of TW on health outcomes, as it is likely to be less contaminated by the pandemic effects, essentially regarding mental health.

### Limitations

A first potential limitation is that our sample is younger than the Portuguese teleworking population, among which 52% are older than 45, for only 20.6% older than 50 in our sample (Data from the National Institute of Statistics, specific module on “work from home”, from 2022, consulted at www.ine.pt on the 2nd of November 2023).

Yet, the education level was quite comparable (74.9% participants with higher education in our sample, for 72.3% in the Portuguese population), such as the occupation type (87.7% working in the services’ sector in our sample, for 85.5% in the Portuguese population. As such, our sample is quite representative of the Portuguese teleworkers, which mostly groups people with high education working in the services sector. Yet, our sample mostly includes teleworkers, which hinders a comparison with people fully working at work premises, including the identification of statistical differences in health outcomes, productivity, and well-being. Second, although the study was carried out in the post-pandemic period, we cannot entirely discard a “pandemic effect”. Unfortunately, no information was collected on the time since TW, which would have helped better disentangle these influences. Third, to increase the sample size, we opted to complete our sample with people to regularly participate in Gfk surveys. The combination of paid and non-paid participants may represent a bias in response behavior, although we did not notice any major difference in the results for each sample considered separately (Table available on demand). Fourth, TW may adopt various models, in terms of location, seasonality, frequency of online contacts, supervision, etc. All these aspects contribute the TW intensity level. In the absence of better information, we limited our analysis to the number of weekly days in TW. This may not fully cover all aspects of the TW intensity but is aligned with most current discussion at firm level, namely about full *versus* hybrid TW, or about the optimal number of TW days in case of a hybrid model.

Finally, the reliance on self-reported data introduces potential sources of bias, including recall bias, justification bias, and desirability bias. For instance, respondents might inadvertently recall or present information in a manner that aligns with their perceptions or preferences, thereby impacting the accuracy of the data collected. Respondents may overestimate the impact of TW on productivity, which stems from a desire to maintain the perceived benefits of their current mode of work. These biases raise concerns regarding the validity of causal inference analyses and the extent to which definitive conclusions can be drawn from our research findings. Given these limitations, while our findings provide valuable insights, it is essential to interpret them with caution and consider the potential influence of the aforementioned biases on the overall outcomes of the study.

### Implications

This paper highlights the relationship between TW conditions, well-being, health, and productivity. That is, even if our sample included people that mostly enjoyed favorable TW conditions, there is a potential harm on health and wellbeing on those who do not enjoy such conditions. This result highlights the need of monitoring TW conditions, and possibly providing support to those who are not expected to benefit from adequate housing conditions.

To our best knowledge, Portugal was the first European country that legally regulated TW in the private sector, in 2003. One of the main objectives of the Portuguese law was to guarantee equal treatment of workers in this regime. In practice, the law defines a list of limited compensation values to be paid to the teleworker (a non-dedicated daily payment). At the organizational level, teleworkers are subject to the same limits of daily and weekly working hours as other workers, while remote meetings and joint tasks must take place within working hours. Nevertheless, in practice, not all these legal concerns are applied, and workers have rarely been compensated.

Based on our results, we suggest that regulation should include a worker medical surveillance, started with a self-report questionnaire, and complemented by a physical examination by an occupational healthcare professional (physician or nurse). Preventive and corrective actions at worker’s home may reveal necessary. All procedures referred should be framed by laws, to ensure that occupational health prevention is effective.

## Conclusions

Most teleworkers highlight a positive perspective about teleworking, enhanced productivity, namely those with favorable working conditions. This global result might be related to the characteristics of our sample, mostly high-educated young adults. Yet, a substantial proportion of our sample report a worsening in health-related behaviors (smoking, alcohol, inadequate diet), self-reported health, and several physical and mental health symptoms and conditions. The link between these unfavorable outcomes and worse TW conditions suggest that support is needed for teleworkers to prevent ill-health conditions. This is the price to pay for teleworking to achieve its maximum benefit in terms of workers’ well-being and productivity. Yet, most participants reported that they received no company support, which may take various forms, from financial support to acquire adapted equipment to monitoring of working conditions at home.

### Supplementary Information

Supplementary Material 1.

## Data Availability

The anonymized database is available upon request, to be addressed to twork4health@ensp.unl.pt.
