# Supplementary material for: Teleworking: does it make workers healthier and productive? A cross-sectional study on a Southern European population
Source: BMC Public Health. 2024 Jul 20;24:1946. doi: 10.1186/s12889-024-19481-y (PMC11264928; doi:10.1186/s12889-024-19481-y)
Supplement: Supplementary file 1 — Supplementary Material 1. [file 12889_2024_19481_MOESM1_ESM.docx]

Supplementary Table S1. Association between covariates and probability to report bad health and productivity worsening (results from logistic regression)

|  | Poor health |  | Productivity worsening |  |
| --- | --- | --- | --- | --- |
|  | Marginal effect | S.E | Marginal effect | S.E |
| Sex (Ref: Female) |  |  |  |  |
| Male | -0.040 | 0.025 | -0.004 | -0.015 |
| Age groups (Ref: <30) |  |  |  |  |
| 30-39 | 0.051 | 0.037 | -0.055 | 0.028 |
| 40-49 | 0.037 | 0.035 | -0.058 | 0.028 |
| 50-59 | 0.068 | 0.044 | -0.046 | 0.031 |
| >60 | -0.001 | 0.064 | -0.015 | 0.056 |
| Education (Ref: primary) |  |  |  |  |
| Secondary | -0.002 | 0.144 | . | . |
| Tertiary | -0.011 | 0.143 | . | . |
| Remote work (Ref: 1-2 days) |  |  |  |  |
| 3-4 days | 0.051 | 0.036 | -0.022 | 0.022 |
| Undefined hybrid | 0.063* | 0.032 | -0.013 | 0.021 |
| Full time | 0.053 | 0.037 | -0.013 | 0.023 |
| Working conditions at home (Ref: no limitation) |  |  |  |  |
| Any limitation | 0.129** | 0.033 | 0.060** | 0.020 |
| Health worsening | . | . | 0.066** | 0.025 |

*p-value<0.05; **p-value<0.01
